# Intrathecal leptin inhibits expression of the P2X_2/3_ receptors and alleviates neuropathic pain induced by chronic constriction sciatic nerve injury

**DOI:** 10.1186/1744-8069-9-65

**Published:** 2013-12-10

**Authors:** Xin Li, Lumei Kang, Guilin Li, Huihong Zeng, Lei Zhang, Xiang Ling, Hui Dong, Shangdong Liang, Hongping Chen

**Affiliations:** 1 Department of Physiology, Medical College of Nanchang University, 603 Bayi Road, Nanchang, Jiangxi 330006, PR China; 2 Department of Animal Science, Medical College of Nanchang University, Nanchang, Jiangxi 330006, PR China; 3 Department of Histology and Embryology, Medical College of Nanchang University, 603 Bayi Road, Nanchang, Jiangxi 330006, PR China; 4 Department of Cardio-thoracic Surgery, The First Affiliated Hospital of Nanchang University, Nanchang, PR China

**Keywords:** Leptin, Dorsal root ganglion, Neuropathic pain, Chronic constriction sciatic nerve injury

## Abstract

**Background:**

Leptin, an adipocytokine produced mainly by white adipose tissue, has a broad role in the regulation of neuronal functions. Accumulating evidence has revealed that leptin plays an important role in influencing neuropathic pain, shown recently by the finding that chronic administration of leptin induced thermal hyperalgesia and mechanical allodynia in naïve rats. Chronic constriction sciatic nerve injury (CCI) is a well characterized model used for studying neuropathic pain. The present study was designed to investigate whether leptin plays a role in neuropathic pain in rats induced by CCI by examining particular pain behaviors.

**Results:**

After sciatic nerve injury in rats, endogenous levels of leptin and leptin receptor (OB-Rb) were increased in a time dependent manner within the ipsilateral dorsal root ganglion (DRG). Intrathecal administration of leptin once daily for 6 days, beginning 7 days after CCI, alleviated neuropathic pain and decreased the expression of IL-6, TNFα, and the P2X_2_ and P2X_3_ receptors. Attenuation of endogenous OB-Rb in the DRG by intrathecal administration of OB-Rb antisense oligonucleotides did not change thermal hyperalgesia or mechanical allodynia induced by CCI.

**Conclusions:**

Our findings suggest that exogenous leptin can alleviate the chronic neuropathic pain caused by CCI. The leptin effect may be mediated by attenuated expression of IL-6, TNFα, and the P2X_2_ and P2X_3_ receptors in the DRG of CCI rats.

## Background

Neuropathic pain is a severe health problem for which there is a lack of effective therapy [1]. It is caused by an injury to the peripheral or central nervous system. Characteristic features of neuropathic pain are hyperalgesia allodynia and spontaneous pain [2]. Numerous neuroanatomical, neurophysiological, and neurochemical mechanisms are thought to contribute to the development and maintenance of neuropathic pain [3]. However, neuropathic pain remains a prevalent and persistent clinical challenge as its pathogenesis is unknown. Consequently, there is a considerable need to explore novel treatment modalities for neuropathic pain management.

The dorsal root ganglion (DRG) is an anatomically discrete structure that forms part of the peripheral nervous system (PNS), and is located laterally to neural tube. It contains pseudounipolar neurons that convey sensory information from the periphery to the central nervous system (CNS). The DRG is recognized as one of the organs that may be damaged in peripheral sensory neuropathic pain states [4]. Furthermore, It has been well established that nerve lesions induce changes in gene and protein expression within the DRG which correspond to induced neuropathic pain [5,6].

Leptin, the product of the obese (ob) gene, is a 16-kDa polypeptide hormone that was first associated with obesity and shown to be secreted by adipose tissue [7]. This initial interest in leptin was concerned with its effects on fat mobilization and energy homeostasis. However more recently, numerous additional roles have been identified, Thus it has been shown that leptin plays a vital role in the regulation of numerous and varied biochemical pathways throughout the body, such as in metabolism, immune and reproductive function, bone homeostasis, insulin sensitivity and neuronal protection. The actions of leptin are mediated by leptin receptors that are widely distributed across many tissues including the spleen, testes, kidney, liver, lung, adrenal, pituitary, hypothalamus and brain. In addition, our previous research found that leptin receptors are expressed in DRG neurons [8].

A variety of studies have shown that leptin plays a pivotal role in neuronal survival and neuroprotection [9,10], and importantly, that acute leptin treatment enhances functional recovery after spinal cord injury [11]. Recent reports also provide a link between leptin and chronic neuropathic pain. Intrathecal leptin administration for 7 days induced thermal hyperalgesia and mechanical allodynia in naïve rats similar to that seen in CCI rats [12]. Leptin-deficient animals, (*ob/ob* mice), showed an absence of tactile allodynia induced by partial sciatic nerve ligation (PSL). However, daily perineural injection of leptin into the ligatured SCN during the early phases of PSL reversed the failure of ob/ob mice to develop tactile allodynia. By contrast, treatment of ob/ob mice with leptin during late phases of PSL did not affect the failure of these mutant mice to develop PSL-induced tactile allodynia [13]. However, under neuropathic pain conditions, the role of leptin is still unknown. In this report we explore the possibility that intrathecal exogenous leptin can alleviate neuropathic pain in a rat model of CCI. We subsequently investigate whether the leptin effect on neuropathic pain we identify is mediated by pain relevant mediators including the P2X_2_ and P2X_3_ receptors.

## Results

### Leptin and OB-Rb are up-regulated in L4-6 DRG of CCI rats

**Figure 1:**
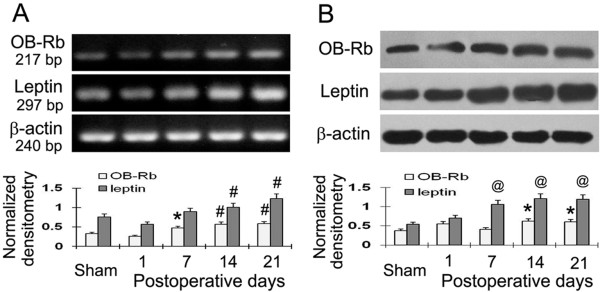
**Leptin and OB-Rb were upregulated in a time dependent manner in L4-6 DRG of CCI rats. (A)** The expression of leptin mRNA and OB-Rb mRNA was increased in the DRG of CCI rats. Leptin and OB-Rb mRNAs were detected by RT-PCR at days 1, 7, 14 and 21 after CCI. **(B)** Leptin and OB-Rb protein were increased in the DRG of CCI rats. Leptin and OB-Rb protein were detected by Western Blot analysis at days of 1,7,14 and 21 after CCI. Data are shown as mean ± S.E.M, n = 6. ^*^P < 0.05, ^#^*P* < 0.01 and ^@^*P* < 0.001 vs sham group.

It has been reported that leptin and leptin receptor (OB-Rb) levels increased in the spinal cord after CCI [12]. However, the expression pattern of leptin and OB-Rb in the ipsilateral DRG of CCI rats is still unknown. In the present study, the time course of the expression of leptin and OB-Rb in the DRG in response to CCI was analysed using semi-quantitative RT-PCR and Western Blot analysis. Leptin and OB-Rb levels were measured at 1, 7, 14 and 21 days after CCI and in the sham group (Figure 1). The results show that both leptin mRNA and protein levels significantly increased 14 and 7 days after CCI respectively, as compared to the sham group. Maybe CCI lead to a response including improved leptin mRNA translation, thus causing leptin protein levels to increase prior to the detected mRNA upregulation. In addition, we found that OB-Rb mRNA and protein levels significantly increased 7 and 14 days after CCI respectively, as compared to the sham group. At day 21 after CCI, leptin and OB-Rb were still maintained at high levels (Figure 1).

### Intrathecal leptin administration alleviated the neuropathic pain of CCI rats

**Figure 2:**
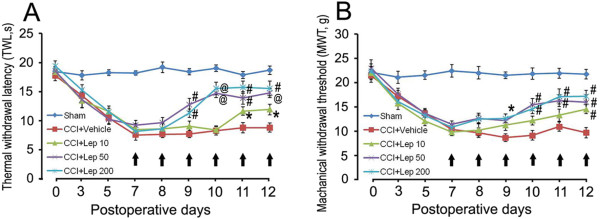
**Leptin alleviates the neuropathic pain.** The thermal withdrawl latency TWL **(A)** and mechanical withdrawl threshold MWT **(B)** were measured after intrathecal administration of leptin. Leptin was intrathecally delivered once daily for 6 days beginning 7 days after CCI, as indicated by arrows. The TWL and MWT were tested 2 h after each administration of leptin. Data are shown as mean ± S.E.M, n = 5. ^*^P < 0.05, ^#^*P* < 0.01 and ^@^*P* < 0.001 vs CCI + Vehicle group.

It has been shown that chronic administration of leptin induced thermal hyperalgesia and mechanical allodynia in the naïve rat model [12]. However, any effect of leptin on thermal hyperalgesia and mechanical allodynia induced by CCI is unknown. In this study, leptin was intrathecally delivered once daily for 6 days beginning 7 days after CCI. The thermal withdrawl latency (TWL) and withdrawal threshold (MWT) were measured to evaluate the effects of leptin on pain thresholds in CCI rats. At day 7 after CCI, a time when neuropathic pain is known to be well established, leptin was intrathecally administered once daily for 6 days. The TWL and MWT were measured 2 h after each treatment. As shown in Figure 2, intrathecal treatment with leptin (10 μg/kg) for 4 and 5 days dramatically increased TWL and MWT as compared to vehicle-treated CCI rats. Intrathecal treatment with leptin (50 μg/kg) for 2 and 3 days significantly increased TWL and MWT as compared to vehicle-treated CCI rats. Intrathecal treatment with leptin (200 μg/kg) for 2 days significantly increased both TWL and MWT as compared to the vehicle-treated CCI rats.

### Intrathecal leptin administration attenuated the expression of IL-6, TNFα, and the P2X_2_ and P2X_3_ receptors in L4-6 DRG of CCI rats

**Figure 3:**
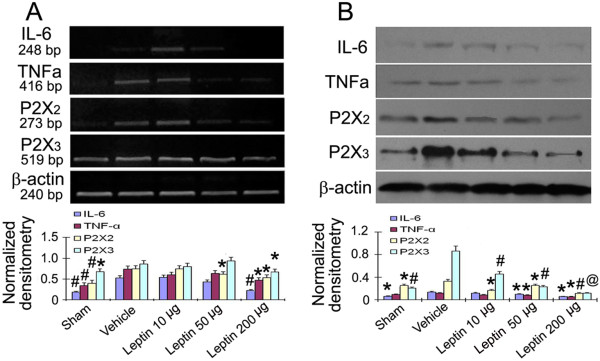
**Leptin attenuates the expression of IL-6, TNFα, and the P2X**_**2 **_**and P2X**_**3 **_**receptors in L4-6 DRG of CCI rats. (A)** Leptin decreased the expression of P2X_2_, P2X_3_ receptors, IL-6 and TNFα mRNAs in a dose dependant manner. Leptin was intrathecally delivered once daily for 6 days beginning 7 days after CCI. The rats were sacrificed 24 h after the last leptin administration. The expression of P2X_2_ and P2X_3_ receptors mRNAs, and IL-6 and TNFα mRNAs were detected using RT-PCR. **(B)** Leptin decreased the expression of P2X_2_, P2X_3_, IL-6 and TNFα protein in a dose dependant manner. Leptin was delivered intrathecally once daily for 6 days beginning 7 days after CCI. The rats were sacrificed 24 hour after the last leptin administration. The expression of P2X_2_, P2X_3_, IL-6 and TNFα protein was detected by Western Blot. Data are shown as mean ± S.E.M, n = 6. ^*^P < 0.05, ^#^*P* < 0.01 and ^@^*P* < 0.001 vs CCI + Vehicle group.

To determine the functional role of the leptin effect on neuropathic pain at a molecular level, we examined whether leptin would modulate the expression of IL-6, TNFα, and the P2X_2_ and P2X_3_ receptors. It has previously been shown that IL-6 protein and mRNA levels increased in both lumbar (L4-5) and cervical (C7-8) dorsal root ganglia (DRG) following CCI [14], and that IL-6 contributes to the development of neuropathic pain following motor fiber injury [15]. Our previous studies have shown that P2X_2/3_ receptors participate in the regulation of neuropathic pain [16]. In the present work, we report that IL-6, TNFα, and the P2X_2_ and P2X_3_ receptors were increased in the DRG of CCI rat (Figure 3). Furthermore, intrathecal administration of leptin (50 μg/kg and 200 μg/kg) efficiently attenuated the expression of IL-6, TNFα, and the P2X_2_ and P2X_3_ receptors (Figure 3). These data indicate that leptin may alleviate the pain behaviors by inhibiting IL-6, TNFα, and the P2X_2_ and P2X_3_ receptors in the DRG of CCI rats.

### Intrathecal leptin administration decreased the immunoreactivity of P2X_2_ and P2X_3_ receptors in L4-6 DRG of CCI rats

**Figure 4:**
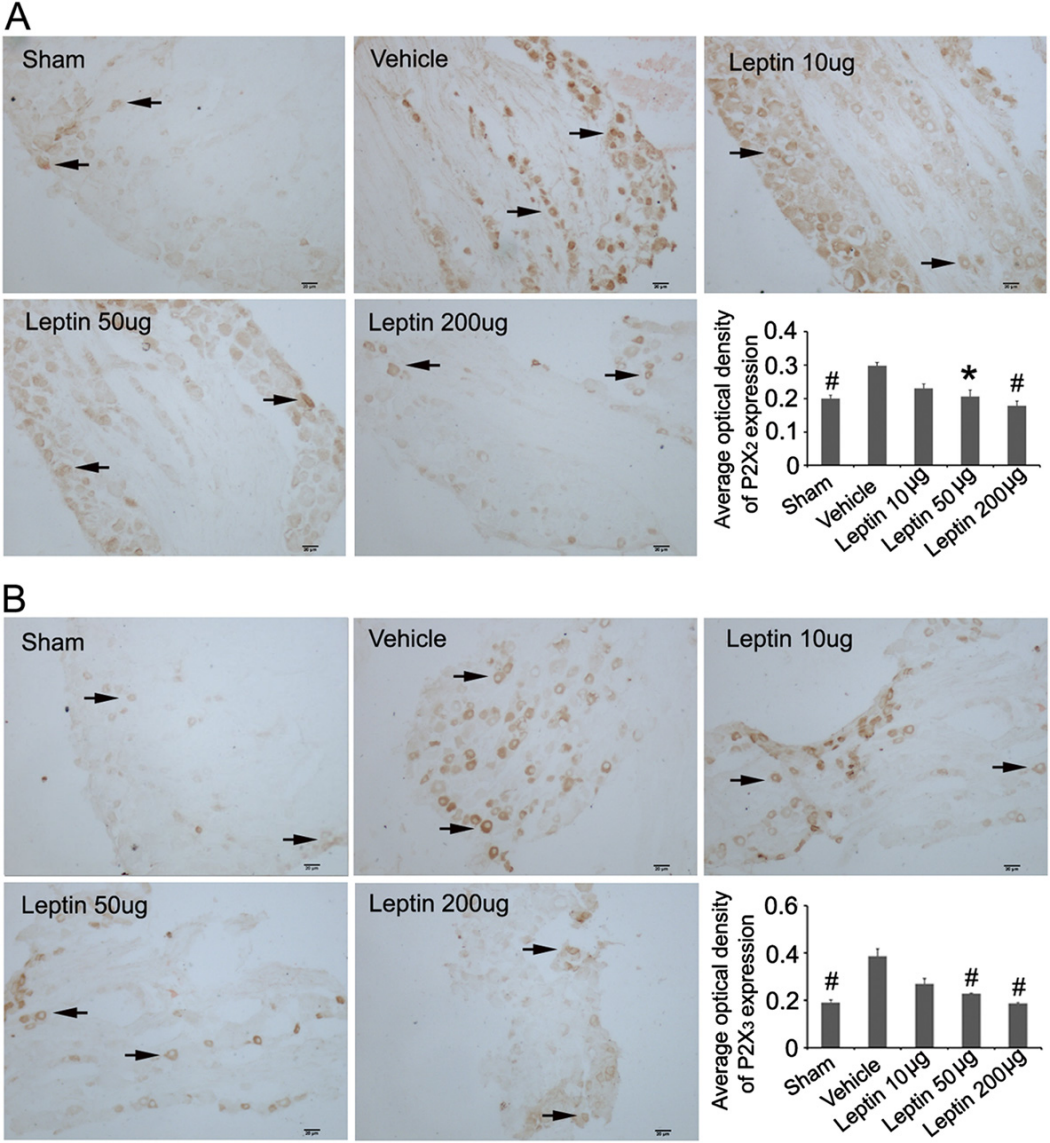
**Leptin administration decreases the immunoreactivities of P2X**_**2 **_**and P2X**_**3 **_**receptors in L4-6 DRG of CCI rats.** Leptin administration decreased the immunoreactivity of P2X_2_**(A)** and P2X_3_**(B)** receptors within the DRG of CCI rats. P2X_2_ and P2X_3_ receptors immunoreactivity (brown) was detected in DRG neurons of sham, CCI + vehicle, CCI + Leptin (10 μg), CCI + Leptin (50 μg) and CCI + Leptin (200 μg) treatments. Leptin was intrathecally delivered once daily for 6 days beginning 7 days after CCI. The rats were sacrificed 24 h after the last leptin administration. Arrows indicate P2X_2_ and P2X_3_ receptor positive neurons. Scale bar is 20 μm. Data are shown as mean ± S.E.M, n = 5. ^*^P < 0.05, and ^#^*P* < 0.01 vs CCI + Vehicle group.

Glia and neurons in the DRG can be clearly distinguished under the light microscope. Immunohistochemical staining using antibodies directed against P2X_2_ and P2X_3_ demonstrated both these receptors werepresent in the cytoplasm of DRG neurons. A semi-quantitative approach was taken to evaluate receptor levels using optical density to generate “stain values” for each group that were subsequently compared. These results indicated that P2X_2_ and P2X_3_ receptor expression increased in DRG neurons of CCI rat at the protein level; furthermore, intrathecal leptin administration (50 μg/kg and 200 μg/kg) significantly attenuated expression of P2X_2_ and P2X_3_ receptors (Figure 4).

### Effects of intrathecal OB-Rb antisense oligonucleotides on the pain behaviors induced in CCI rats

**Figure 5:**
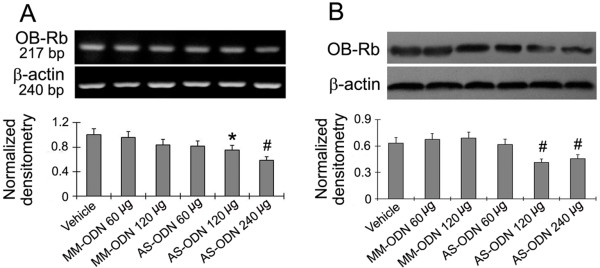
**Intrathecal administration of OB-Rb antisense oligonucleotides decreased OB-Rb mRNA and protein expression. (A)** Intrathecal OB-Rb antisense oligonucleotides (120 μg/kg and 240 μg/kg) significantly decreased the expression of OB-Rb mRNA. **(B)** Intrathecal OB-Rb antisense oligonucleotides (120 μg/kg and 240 μg/kg) significantly decreased the expression of OB-Rb protein. The drugs were delivered intrathecally once daily for 6 days beginning on day 7 after CCI. The rats were sacrificed 24 h after of the last leptin administration. Data are shown as mean ± S.E.M, n = 6. ^*^P < 0.05, and ^#^*P* < 0.01 vs CCI + Vehicle group.

**Figure 6:**
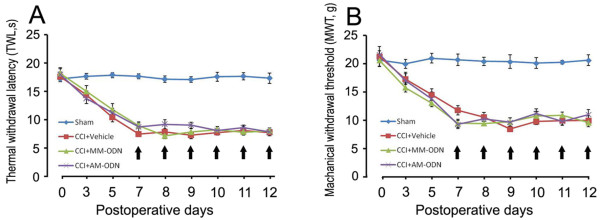
**Effects of intrathecal OB-Rb antisense oligonucleotides on the pain behavior induced in CCI rats.** The thermal withdrawl latency TWL **(A)** and mechanical withdrawl threshold MWT **(B)** were measured after intrathecal administration of antisense nucleotides directed against the leptin receptor. The AS oligonucleotides were delivered intrathecally once daily for 6 days beginning on day 7 after CCI, as indicated by arrows. The TWL and MWT were tested 2 h after each administration of leptin. Data are shown as mean ± S.E.M, n = 6.

It has been reported that deletion of the leptin receptor in mice prevented neuropathic pain development [17]. However, the role the leptin receptor signal plays in neuropathic pain is still unknown. Therefore, we evaluated whether there was any change in the pain behaviors TWL and WMT in CCI rats following intrathecal administration of OB-Rb antisense oligonucleotides. We found that OB-Rb antisense oligonucleotides (120 μg/kg and 240 μg/kg) significantly attenuated the expression of OB-Rb (Figure 5). However, OB-Rb antisense oligonucleotides (240 μg/kg) could not change the pain behaviors induced in the CCI model (Figure 6).

## Discussion

The present study demonstrated the following novel findings: (1) leptin and OB-Rb are expressed within the ipsilateral DRG and are up-regulated after CCI in a time-dependent manner. (2) Exogenous leptin administration alleviated the chronic neuropathic pain of rat caused by CCI. (3) Exogenous leptin administration attenuated the expression of IL-6, TNFα, and the P2X_2_ and P2X_3_ in rat DRG induced by CCI. (4) Attenuation of endogenous OB-Rb expression in the DRG by intrathecal OB-Rb antisense oligonucleotides did not change the thermal hyperalgesia or mechanical allodynia induced by CCI. These results reveal a critical role of leptin in neuropathic pain and a functional link between leptin and P2X_2/3_ receptors, IL-6 and TNFα. These findings suggest a different role for leptin in CCI rats compared to naïve rats.

Leptin is known to influence brain development. Leptin deficient ob/ob mice have smaller brains [18] and leptin administration increased brain weight in ob/ob mice [19]. It has also been shown that leptin can play a neuroprotective role after neuronal injury. Leptin protects against delayed ischemic neuronal death in hippocampal CA1 neurons by maintaining the pro-survival states of the Akt and ERK1/2 MAPK signaling pathways, thereby preventing apoptotic neuronal loss [20]. Leptin has a prominent neuroprotective and anti-inflammatory role following spinal cord damage and together these studies highlight leptin as a promising therapeutic agent [11]. Administration of leptin to transgenic mouse models of AD (Alzheimers disease) reduces neuronal pathology and improves cognitive performance [21].

Recent studies have shown leptin plays an important role in neuropathic pain induced by nerve injury. Chronic administration of leptin induced thermal hyperalgesia and mechanical allodynia in naïve rats [12] and its mechanism involved an enhancement of N-methyl-_D_-aspartate (NMDA) induced spinal excitation [22]. Interestingly, leptin administration afforded significant neuroprotection of mouse cortical neurons against NMDA cytotoxicity [23]. These results suggest that leptin contributed towards neuropathic pain through evoking NMDA signaling in naïve rats but alternatively, performed a neuroprotective role by inhibiting NMDA cytotoxicity under conditions of nerve injury. Thus, we evaluated the role(s) of leptin on neuropathic pain induced by CCI in rats. Our results show that exogenous leptin administration alleviated the pain behaviors induced by CCI. The mechanism of this action may be relevant to the neuroprotective role of leptin under conditions of nerve injury. However, decreasing the OB-Rb levels in the DRG of CCI rat did not change the TWL and MWT pain behaviors.

Adenosine, 5′-triphosphate (ATP) is a ubiquitous molecule found in every cell in the millimolar concentration range, and is released into the extracellular matrix after tissue injury. ATP release from different cell types is implicated in the initiation of pain by activating P2 receptors on sensory nerve terminals [24]. Known P2X subtypes with a role in nociception include P2X_3_ and P2X_2/3_ receptors, which are considered potential therapeutic targets for the management of pathological conditions. Suppressing the expression of P2X_3_ receptors in the DRG, attenuated hyperalgesia following CCI in rats [25]. Activation of the P2X_3_ receptors produced fast desensitizing currents in DRG neurons, and in contrast, P2X_3_ mouse mutants showed either a lack of fast desensitizing currents induced by ATP or a significant reduction in pain behaviors in response to ATP [26,27]. Our previous results similarly showed that inhibiting the P2X_2/3_ receptors of primary sensory neurons alleviated chronic neuropathic pain [28]. In this study, we found that leptin could alleviate the pain behaviors induced by CCI and decreased the expression of P2X_2/3_ receptors. These results suggest that the effect of leptin on neuropathic pain is partly mediated by inhibiting the expression of P2X_2/3_ receptors.

Neuropathic pain often develops following peripheral nerve damage. In such pathological conditions, proinflammatory cytokines and chemokines are upregulated in the DRG associated with the injured nerves [29]. Increasing IL-6 levels in afferent neurons in the DRG and spinal cord contributes to the development of neuropathic pain following motor fiber injury [15]. IL-6 protein was significantly elevated in DRG of CCI rats in a time dependant manner [30]. Interestingly, the IL-6 signal is also involved in the maintenance of experimentally induced mechanical hypersensitivity [31]. Several lines of evidence indicate that TNFα also plays a key role in neuropathic pain. In response to either peripheral nerve injury or after spinal cord injury, TNFα levels are increased in the spinal cord [32,33]. In the CCI model of peripheral neuropathic pain, neutralizing antibodies directed against TNFα reduce thermal hyperalgesia and mechanical allodynia [34]. In this study, we found that administration of leptin decreases the expression of IL-6 and TNFα. These results imply that the effect of leptin on alleviating neuropathic pain is partly mediated by inhibiting the expression of IL-6 and TNFα.

The actions of leptin are mediated by its receptor OB-Rb. OB-Rb exists in a number of different isoforms which are distinguished by the length of their intracellular domains as the long isoform (OB-Rb) and short isoforms (OB-Rs). The OB-Rb isoform is believed to be the functional signal-transducer in the hypothalamus, while the remaining OB-Rs are thought to serve as leptin transporters or to mediate leptin degradation [35]. Mice with the leptin receptor null mutation (db/db) demonstrate a decreased sensitivity to mechanical stimulation and a decreased nociceptive response in the affected hind paw during the second phase of a formalin test [17]. In the current study, our results show that attenuation of endogenous OB-Rb expression by intrathecal OB-Rb antisense oligonucleotides did not change thermal hyperalgesia or mechanical allodynia induced by CCI. Together these findings suggest that blocking the leptin receptor prevents neuropathic pain development. However, during the CCI pain condition, attenuation of OB-Rb expression did not change the TWL and MWT, easily identifiable pain behaviors. Therefore, the mechanism underlying alleviation of neuropathic pain by leptin is unknown and needs to be further investigated in order to provide options for the treatment of pain.

## Conclusions

In conclusion, our study demonstrates that leptin and OB-Rb were increased within the DRG after sciatic nerve injury. Intrathecal leptin alleviated neuropathic pain and decreased the expression of P2X_2_ and P2X_3_ receptors, IL-6 and TNFα. Attenuation of the OB-Rb in the DRG of CCI rats did not change the pain behaviors. These data illustrate that leptin can alleviate chronic neuropathic pain, and this role of leptin may be mediated by P2X_2_ and P2X_3_ receptors, IL-6 and TNFα.

## Materials and methods

### Animals

Male Sprague-Dawley rats weighing 220-250 g were provided by the Center of Laboratory Animal Science of Nanchang University. The rats were fed a standard laboratory diet under controlled temperature and 12-h light/dark cycle at 20-22°C. All experimental procedures were approved by the Institutional Animal Care and Use Committee of the Medical College of Nanchang University. All efforts were undertaken to minimize the number of animals used and their discomfort.

### Experimental design

Four series of experiments were performed in this study. In the first experiment, to analyze the time course of expression of leptin and OB-Rb in rat DRG after CCI, rats were randomly divided into 5 groups with 6 rats in each group: a sham group (Sham), a postoperative day 1 group (D1), a postoperative day 7 group (D7), a postoperative day 14 group (D14) and a postoperative day 21 group (D21). Expression levels of leptin and OB-Rb in L4-6 DRG were analysed at days 1, 7, 14 and 21 after CCI for the experimental groups D1, D7, D14 and D21 described above. For the sham group, operations were performed and DRGs harvested and analysed for OB-Rb and leptin on day 7.

In the second experiment, to evaluate the role of leptin on neuropathic pain, and P2X_2_ and P2X_3_ receptors, IL-6 and TNFα expression, rats were randomly divided into 5 groups with 8 rats in each group: a sham group (sham), a vehicle group (Vehicle), and three experimental groups with leptin administration at 10 μg/kg (leptin 10 μg), at 50 μg/kg (leptin 50 μg) and 200 μg/kg (leptin 200 μg). The drugs were delivered intrathecally once daily for 6 days, beginning on day 7 after CCI.

In the third experiment, to test the effects of inhibiting OB-Rb expression using OB-Rb antisense oligonucleotides, rats were randomly divided into 6 groups with 6 rats in each group: a vehicle group (Vehicle), as well as two control groups of rats with mismatch oligonucleotides to OB-Rb at 60 μg/kg (MM-ODN 60 μg), and 120 μg/kg (MM-ODN 120 μg). There were three experimental groups with OB-Rb antisense oligonucleotides administered at 60 μg/kg (AS-ODN 60 μg), 120 μg/kg (AS-ODN 120 μg) or or240 μg/kg (AS-ODN 240 μg). The drugs were delivered intrathecally once daily for 6 days beginning on day 7 after CCI.

In the fourth experiment, to examine the role of OB-Rb antisense oligonucleotides on pain behaviors induced by CCI, the rats were randomly divided into 4 groups with 6 rats in each group: a sham group (sham), a vehicle group (Vehicle), a group administered mismatch oligonucleotides at 120 μg/kg (MM-ODN 120 μg) and a group administered OB-Rb antisense oligonucleotides at 240 μg/kg (AS-ODN 240 μg). The drugs were delivered intrathecally once daily for 6 days, beginning on day 7 after CCI.

### The chronic constriction injury (CCI) model

The CCI model was established as previously described [36]. Briefly, rats were anesthetized with sodium pentobarbital (40 mg/kg, i.p.). The sciatic nerve was exposed and loosely ligated with sterile 4-0 catgut thread at four consecutive sites with an interval of approximately 1 mm. Meanwhile, a sham surgery was performed with the sciatic nerve exposed but not ligated. Animals were kept warm and allowed to recover from anaesthesia.

### Evaluation of the pain behavior

The testing procedure was performed according to previously published protocols [36]. The mechanical withdrawal threshold (MWT) was determined to evaluate mechanical hyperalgesia using calibrated von Frey filaments (BME-403, Tianjing, China). Thermal hyperalgesia was measured using a thermal paw stimulation system (BME-410C, Tianjin, China) and expressed as thermal withdrawal latency (PWL), the time taken for thermal discomfort to be noticed and elicit paw withdrawal. Each rat was measured three times and the mean value was taken as the threshold value.

### Reverse transcription-polymerase chain reaction (RT-PCR)

Total RNA was isolated using TRIzol (Invitrogen, Carlsbad, CA, USA) according to the manufacturer’s protocol. 1000 ng total RNA was used as a template for reverse transcription using the Applied Biosystems Reverse Transcription Kit (Applied Biosystems, Foster City, CA, USA). PCR amplification of P2X_2_, P2X_3_ receptors and β-actin (control) was carried out according to our previous method using olignucleotides as described in [28]. Oligonucleotides for amplification of leptin and OB-Rb were as follows: for leptin, sense: 5′-CCTGGAAGCCTCGCTCTACT-3′, and antisense: 5′-ATGGAATCGTGCGGATAACT-3′; for OB-Rb, sense: 5′-CTGGGTTTGCGTATGGAAGT-3′, and antisense: 5′-CCAGTCTCTTGCTCCTCACC-3′. The PCR products were amplified using the following cycling parameters: 94°C for 5 min, followed by 35 cycles of 94°C for 45 s, 53°C for 30 s, and 72°C for 40 s, and finally a single cycle at 72°C for 5 minutes.

### Western blot analysis

30 μg samples of total protein were separated using 6.5% (for OB-Rb analysis) or 10% (for P2X_2_ and P2X_3_ receptors, leptin and IL-6 analysis) SDS-polyacrylamide gel electrophoresis and transferred onto a polyvinylidene difluoride membrane. After incubation with primary antibody against either P2X_2_ or P2X_3_ receptors, IL-6, leptin or OB-Rb (Santa Cruz Biotecnology, Santa Cruz, CA, USA), the membrane was incubated with peroxidase conjugated secondary antibodies (Cell Signaling Technology, Danvers, MA, USA). Immunodetection was by using the Pierce-enhanced chemiluminescence substrate (Thermo Scientific, Waltham, MA, USA). β-actin antibody (Santa Cruz Biotecnology, Santa Cruz, CA, USA) was used as a loading control.

### Immunohistochemistry

Six DRGs from 6 rats were analysed from each group. Formalin-fixed, paraffin-embedded tissue was sectioned at 5 μm, and every fifth section was used for immunohistochemistry (IHC), with at least 40 sections analysed in each group. IHC was carried out as previously described [23]. Primary antibody against P2X_2_ or P2X_3_ receptor (Santa Cruz Biotecnology, Santa Cruz, CA, USA) was used. Protein localization was detected following incubation with diaminobenzidine (DAB) and H_2_O_2_ for 2 min. Finally, sections were dehydrated in ethanol and mounted with neutral balsam.

### Statistical analysis

Data reflect the mean ± S.E.M. Comparisons of means between two groups was carried out using a *t*-test and those between multiple groups with one way analysis of variance (ANOVA). A value of *P* < 0.05 was considered statistically significant.

## Abbreviations

CCI: Chronic constriction sciatic nerve injury; TWL: Thermal withdrawal latency; MWT: Mechanical withdrawal threshold; DRG: Dorsal root ganglion; MM-ODN: Mismatch oligonucleotides; AS-ODN: Antisense oligonucleotide; PSL: Partial sciatic nerve ligation; ATP: Adenosine, 5′-triphosphate.

## Competing interests

The authors declare that they have no competing interests.

## Authors’ contributions

XL, LMK, GLL and HHZ carried out the experiment and analyzed the data. LZ, XL and HD together conceived the study, and participated in its design. SDL coordinated and supervised the experiments. HPC supervised the experiments and wrote the manuscript. All authors have read and approved the final version of the manuscript.

## Authors’ information

Xin Li, Lumei Kang, Guilin Li, Huihong Zeng are senior authors.
